# Gross genetic alterations and genetic heterogeneity in a periductal stromal tumor of the breast

**DOI:** 10.1186/s13039-020-00516-z

**Published:** 2020-11-23

**Authors:** Carsten Holzmann, Burkhard Helmke, Joern Bullerdiek

**Affiliations:** 1 Institute of Medical Genetics, University Rostock Medical Center, Ernst-Heydemann-Str. 8, 18057 Rostock, Germany; 2 Institute of Pathology, Elbe Kliniken, Klinikum Stade, Bremervörder Str. 111, 21682 Stade, Germany; 3 Human Genetics, University of Bremen, 28359 Bremen, Germany

**Keywords:** Periductal stromal tumor, Biphasic breast tumors, Genetic heterogeneity, Pseudotetraploidy, Chromosome 1, Chromosome 5, Chromosome 6

## Abstract

**Background:**

Periductal stromal tumors of the breast are exceedingly rare biphasic breast tumors with close morphological relationship to phyllodes tumors. So far, results of genetic analyses on these tumors have not been reported.

**Case presentation:**

A 50 year old female patient was admitted to the hospital because of a palpable lump in her right breast with a diameter of approximately 5–6 cm which was surgically removed by lumpectomy. Histologic examination revealed a biphasic breast tumor classified as periductal stromal tumor. Array analysis showed a pseudotetraploid tumor with a copy number of 4 for most of the chromosomes. In addition, further changes of chromosomes 1, 5, and 6 were noted but there were no mutations of *MED12* as those frequently seen in fibroadenomas or phyllodes tumors.

**Conclusions:**

The genetic alterations observed indicate karyotypic evolution leading to marked heterogeneity which fits with the tumor´s histologic and cytologic appearance as well as with its malignant behavior. Because of the absence of genetic similarities with phyllodes tumors, the case does not offer evidence for a common entity but rather suggests the existence of two independent entities.

## Background

Periductal stromal tumors of the breast are an exceedingly rare tumor entity with close morphological relationship to phyllodes tumors [1]. Formally, they belong to the myoepithelial tumors and are composed of benign ductal elements and a stroma composed of spindle cells without showing the typical leaf-like histological patterns seen in phyllodes tumors (PT). Due to their rareness, to the best of our knowledge genetic studies of these tumors have not been reported as yet. Herein, we have used genomic arrays to characterize genetically one such tumor. The results are compared with those obtained on PT and with sarcomas in general.

## Materials and methods

All methods described herein are identical to those as described by Holzmann et al. [2].

### Histological examination

For diagnostic purposes tumor samples were fixed in paraformaldehyde (4% in PBS) and processed for paraffin embedding. Tissue sections (1–2 μm) were deparaffinized in xylene, rehydrated through a series of ethanol, and stained with hematoxylin and eosin (H&E) for histological examination.

### DNA isolation and quantification

DNA from an FFPE sample was isolated using the QIAamp DNA Mini Kit (Qiagen, Hilden, Germany) on a QIACube (Qiagen), according to the manufacturer’s instructions. The amount of double-stranded DNA was measured using the Qubit dsDNA HS Assay Kit and a Qubit Fluorometer (Life Technologies, Carlsbad, CA, USA).

### *MED12* mutation analysis

For PCR amplification 1,000 ng of genomic template DNA were used. Primers to amplify the desired human PCR fragment of the *MED12* gene were those recently described [3, 4]. Subsequently, PCR-products were separated by agarose gel-electrophoresis and the desired DNA fragments/bands were extracted by a QIAquick Gel Extraction Kit (Qiagen) using a QIACube (Qiagen) according to manufacturer’s instructions. DNA sequencing of the purified PCR-products was performed by GATC Biotech (Konstanz, Germany).

### MIP assay and array hybridization

The OncoScan FFPE Assay (Affymetrix, Santa Clara, CA) is based on Molecular Inversion Probe (MIP) technology and offers the detection of genome-wide copy number and copy-neutral LOH. More than 330,000 MIPs result in a 300-kb genome-wide copy number resolution and an enhanced copy number resolution of 50–100 kb around approximately 900 cancer genes. Labelling of 80 ng dsDNA and array hybridization was performed following the manufacturer’s instructions. After staining and washing using a GeneChip Fluidics Station 450 (Affymetrix) the arrays were scanned by an Affymetrix 3000 7G scanner. Arrays were analyzed through the Nexus Express Software for OncoScan (BioDiscovery, El Segundo, CA, USA).

### Case presentation

A 50 year old female patient was admitted to the hospital because of a palpable lump in her right breast with a diameter of approximately 5–6 cm. The tumor that was initially classified as a fibroadenoma based on a needle core biopsy was surgically removed by lumpectomy. Because of close margins, reoperation was performed.

**Fig. 1:**
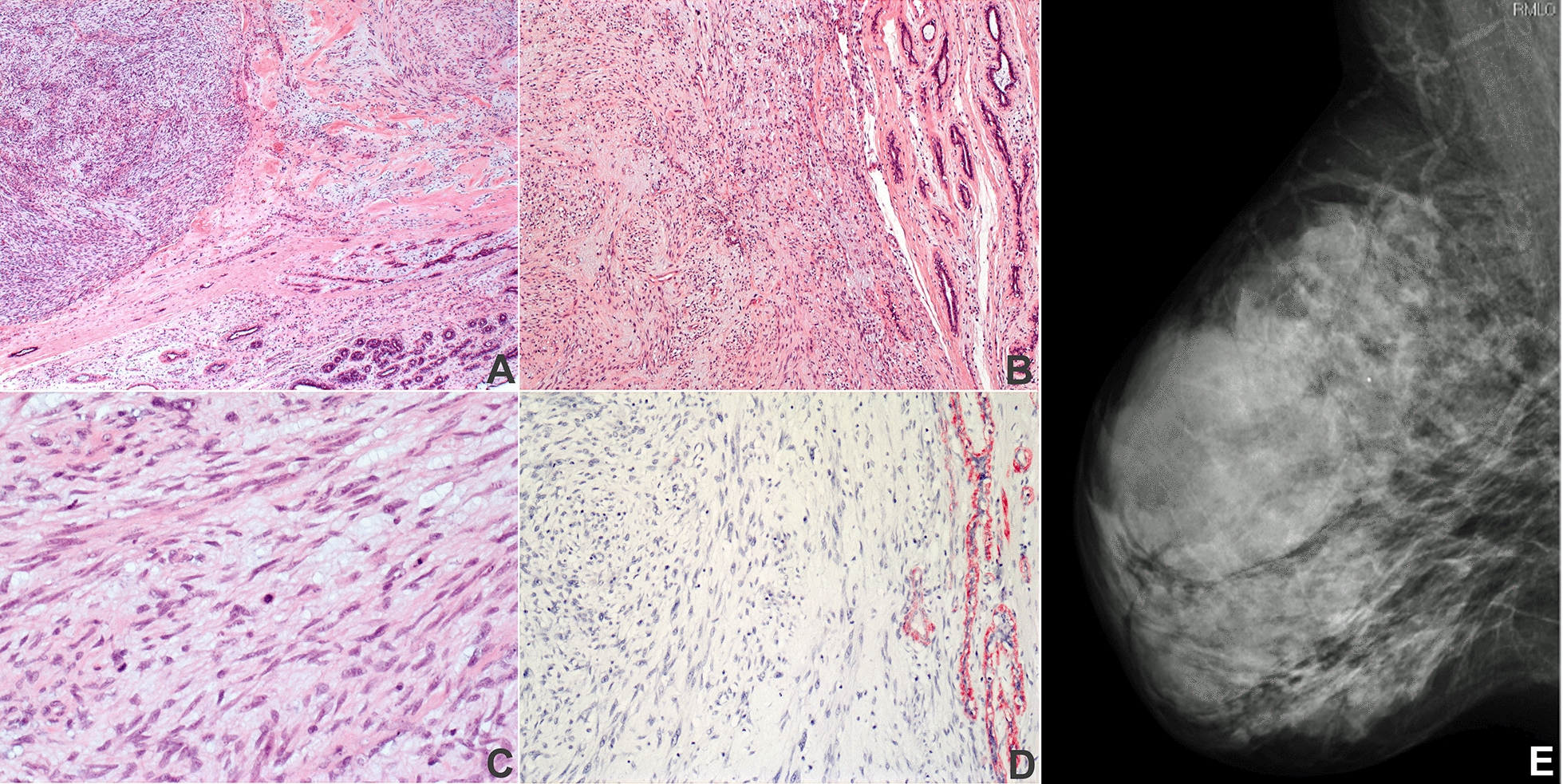
Microscopic and mammographic appearance of the tumor.** A**–**C** HE staining at different magnifications with several mitotic figures that can be seen in **C**, **D** immunostaining for actin, and **E** showing the apparently well-circumscribed tumor at mammography

Histologic examination of the excised tumor showed the pattern of a biphasic tumor in the absence of the characteristic leaf-like patterns of phyllodes tumors or necrotic areas. It was composed of nodules with partially well-defined but focally permeative borders thus excluding typical features of a fibroadenoma. Spindle-shaped cells were embedded in a hyalonous or myxoid stroma surrounding open tubules (Fig. 1a, b). Mitotic activity varied across the lesion with a maximum of 13 mitotic figures per 10 HPF (high power fields) (Fig. 1c) occasionally presenting with atypia. The nuclei of the cells appeared to be of highly variable size and pleomorphism. Very rarely, multinucleated cells were seen as well (Fig. 1c). By immunohistochemistry the expression of vimentin and S 100 and focally of calponin and p63 was demonstrated. In contrast, no expression of either actin, CK 5/6, CK 8/18, nor CD 10 was noted (Fig. 1d). Accordingly, the tumor (Fig. 1e) was classified as a periductal stromal tumor.

After reoperation, extended adenosis was found in the marginal zone of the initially resected tumor. After regular follow-up the patient was found to be recurrence free for more than 36 months.

**Fig. 2:**
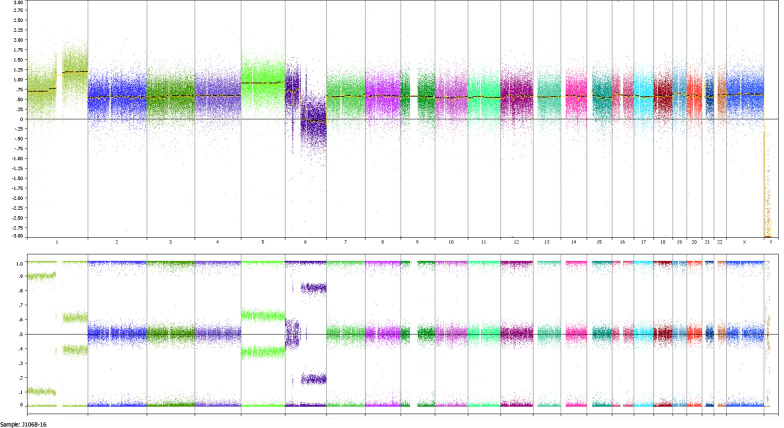
Genomic overview of the CNV array results for the periductal stromal tumor of the breast showing almost whole genome duplication along with further aberrations of chromosomes 1, 5, and 6. Top panel gives the copy number probe intensity calls and the bottom panel displays the calculation of B-allele frequencies (BAF)

Only paraffin-embedded samples of the tumor were available for analysis. Thus, classical analysis was not performed. Overall, array analysis reveals a pseudotetraploid tumor with a copy number of 4 for most of the chromosomes and a 50% AB frequency of the respective BAF plot (Fig. 2). Nevertheless, additional changes of chromosomes 1, 5, and 6 were noted.

**Fig. 3:**
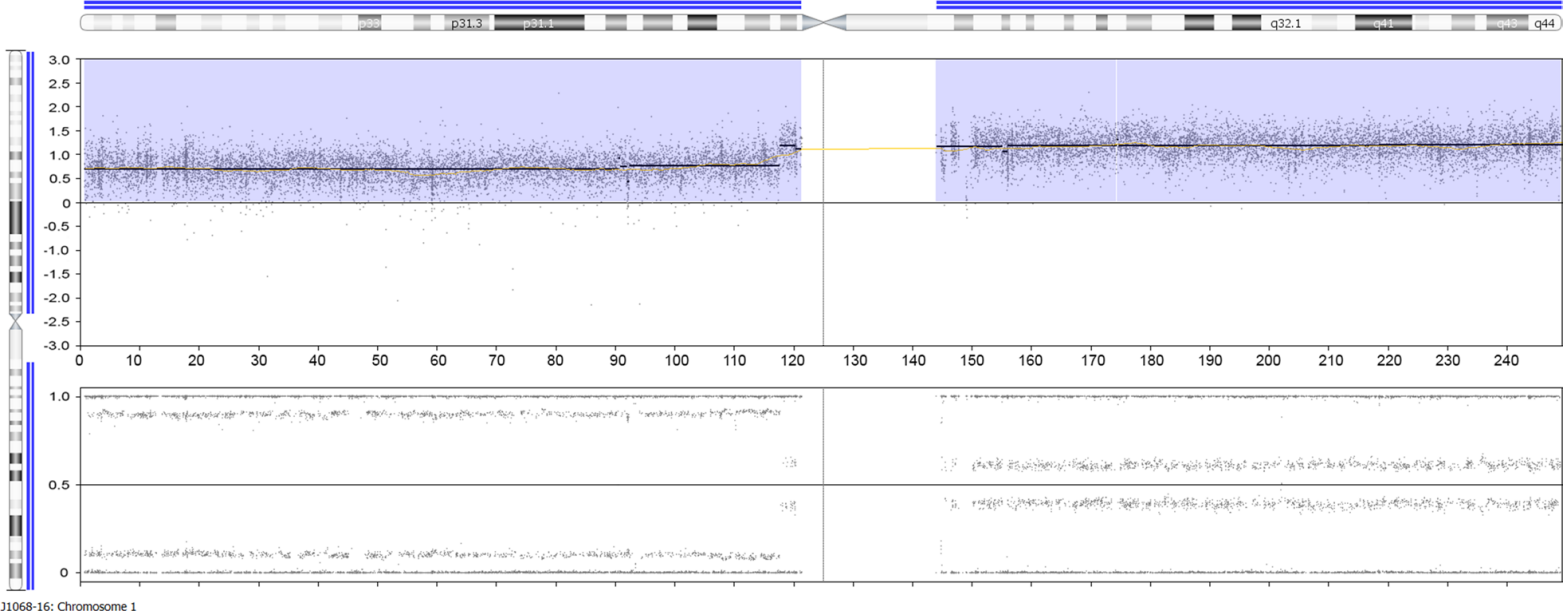
In depth CNV array results for chromosome 1 showing pentasomy of almost the entire short arm and octosomy of the remaining part of it adjacent to the centromere as well as the entire long arm (1p13-1qter). Top panel gives the copy number probe intensity calls and the bottom panel displays the calculation of BAF

As to chromosome 1, almost the entire short arm showed pentasomy while a short part of it adjacent to the centromere as well as the entire long arm (1p13-1qter) was found to be octosomic (Fig. 3) the latter suggesting an origin of the aberration prior to the duplication of the whole chromosome complement. Accordingly, this aberration is also most frequently found compared to the other aberrations as revealed by the BAF plot thus as well offering evidence for its early occurrence during tumorigenesis.

**Fig. 4:**
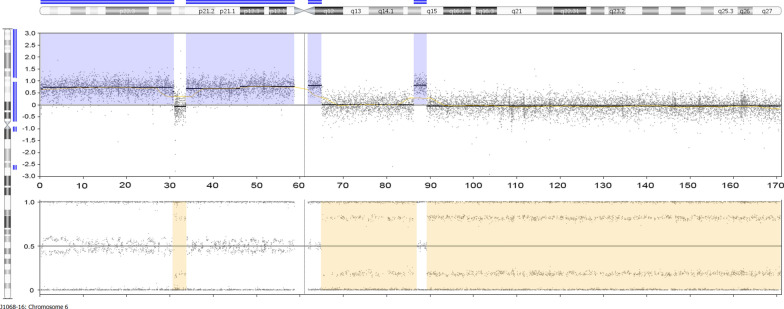
In depth CNV array results for chromosome 6. A short segment of its p-arm as well as almost the entire q-arm were found to be disomic whereas the largest part of the p-arm as well as a short segment of the q-arm adjacent to the centromere as well as a distinct short segment of 6q14-15 were pentasomic. Top panel gives the copy number probe intensity calls and the bottom panel displays the calculation of BAF

The entire chromosome 5 was hexasomic most likely indicating a duplication before duplication of the whole chromosomal complement. As to chromosome 6 data revealed more complex chromosomal alterations (Fig. 4). A short segment of its p-arm as well as almost the entire q-arm were found to be disomic whereas the largest part of the p-arm as well as a short segment of the q-arm adjacent to the centromere as well as a distinct short segment of 6q14-15 were pentasomic. However, these aberrations of chromosome 6 are the second most prevalent among the tumor population although they are apparently not shared by all cells showing aberrations of chromosome 1.

Chromosomes 2–4 and 7–22, respectively, did not show any deviations except for tetrasomy with a copy number of 4 and a 50% AB frequency as indicated by the BAF plots.

In general, genetic heterogeneity among the tumor cell population indicates karyotype evolution during the growth of the tumor.

**Fig. 5:**
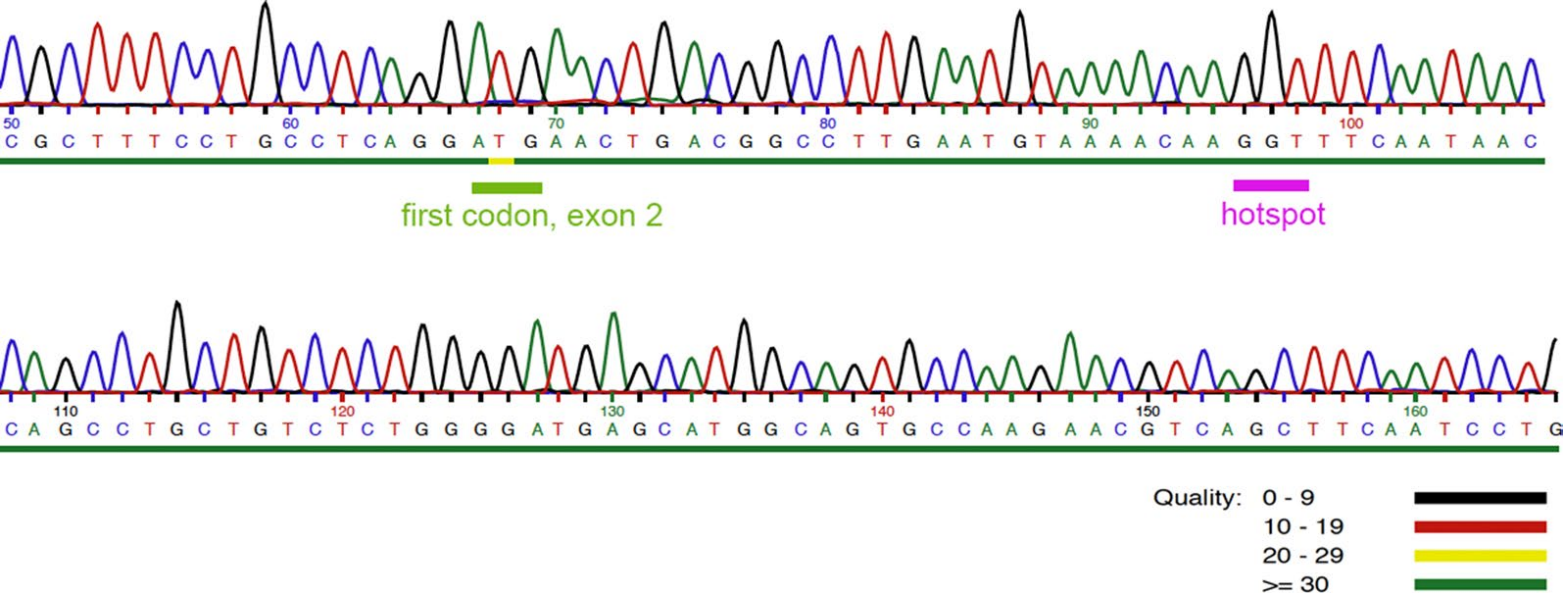
*MED12*, partial sequence including the intron 1-exon 2 boundary and part of exon 2

Because of the high prevalence of *MED12* mutations in phyllodes tumors we have next sequenced the hotspot of these mutations. As a result, no mutations of *MED12* as seen in large subsets of fibroadenomas and phyllodes tumors of the breast were detected (Fig. 5).

## Discussion

Akin to fibroadenomas (FA), hamartomas and phyllodes tumors (PT), periductal stromal tumors of the breast are biphasic breast tumors composed of mesenchyme-derived as well as of epithelial cells forming ducts. By their at least local invasive growth they can be distinguished from FA but they are also lacking the leaf-like growth patterns that characterizes PT and coined their name (phyllodes = greek for leaf). Nevertheless, it has been a matter of debate whether or not periductal stromal tumors represent a rare variant of PT belonging to the same family of biphasic tumors. Alternatively, they are now thought to display histologic features that warrant to establish an own independent entity. Of course, a genetic characterization and comparison with PT may be of valuable help to answer this question but so far, to the best of our knowledge, no results of genetic analyses of periductal stromal tumors of the breast have been reported. Herein, we have applied genomic arrays to perform such analysis.

In general, the genetic alterations observed in the tumor presented herein indicate karyotypic evolution with marked heterogeneity as revealed by investigations using genomic arrays (cf. Fig. 2, BAF lane) the latter being compatible with enhanced growth [5]. A prominent feature of the tumor analyzed here is the duplication of almost the entire genome leading to tetrasomy for all but three chromosomes. Whole genome duplication is occasionally seen in sarcomas in general [6] and has been observed in other malignancies as well. It may lead to a tetrapoloid intermediate (for review: [7, 8]) and be of therapeutic relevance [8, 9]. In non-uterine LMS whole-genome duplication appears to correlate with worse prognosis if associated with gain of 5p14-pter [10] and of note, both these cytogenetic deviations are seen also in the tumor described here. Nevertheless, to the best of our knowledge, whole genome duplication has not been reported in phyllodes tumors or fibroadenomas so far.

Analyses of FA and PT using genomic arrays have been performed previously. Laé et al. [11, 12] have studied 53 borderline (30) as well as malignant (23) phyllodes tumors for chromosome copy number variations (CNVs). Of these, 45 (85%) showed CNVs. In the latter study, the average number of CNA was 3.0. As to the case presented here, given that almost the entire genome of the tumor has been duplicated, copy numbers above 4 are considered reflecting additional gains which were identified for chromosomes 1, 5, and 6, respectively. The highest copy number (8N) was found for the entire long arm of chromosome 1 and a short segment of its short arm immediately adjacent to the centromere suggesting the existing of a deleted derivative chromosome 1 with a short arm deletions existing in four copies. In the study by Laé et al. [11], gains of the long arm of chromosome 1 had been detected in 40% of the tumors (21/53). In an earlier paper, Jardim et al. [13] applied genomic arrays to study a malignant phyllodes tumor. In this case as well, a segment of the long arm of 1q (1q23.3–1q31.2) was found to be amplified. While as to this long arm amplification a similarity with phyllodes tumors becomes apparent, these gains lack specificity since they have been found in a huge variety of solid tumors as well as leukemias. Similarly, deletions of the long arm of chromosome 6 since long have been identified in subsets of a variety of malignant tumors [14]. Tibiletti et al. [15] found deletion of the long arm of chromosome 6 also in a majority of FA investigated but these findings have not been confirmed so far.

Like FA, PT are often showing mutations of the gene encoding mediator complex, subunit 12 (*MED12*) [12, 16]. Most of these aberrations are single nucleotide exchanges or small deletions residing in exon 2 of the gene or the intron 1-exon 2 boundary and. Of note, these mutations are not exclusively found in tumors of the breast but also characterize subsets of benign and malignant uterine smooth muscle tumors [3, 4, 17] and a small group of CLL [18]. The present tumor was shown to lack mutations in the corresponding hotspot region of *MED12*.

Of note, there were also no small deletions of the *HMGA1* and *HMGA2* loci indicating rearrangements of these genes or chromosomal bands 6p21 or 12q14-15, respectively, as described in other biphasic breast tumors [19–24].

In summary, this is the first description of genetic alterations in a periductal stromal tumor. It does not reveal apparent genetic alterations indicating its similarity with fibroadenomas or phyllodes tumors. Because of the absence of such shared similarity, the case does not offer evidence for a common entity between periductal stromal tumors and PT but rather supports the idea of two independent entities. The genetic heterogeneity as detected herein fits with the tumor´s histologic and cytologic appearance as well as with its malignant behavior.

## Data Availability

Details of all data obtained by array CGH are available from the corresponding author upon request.

## Abbreviations

BAF: B-allele frequency
CLL: chronic lymphocytic leukemia
CNA: copy number alteration
CNV: copy number variation
FA: fibroadenoma
FFPE: formalin-fixed paraffin-embedded
HPF: high power field
*MED12*: gene encoding Mediator Complex Subunit 12
MIP: molecular inversion probe
PCR: polymerase chain reaction
PT: phyllodes tumor
